# Continuing the conversation: a cross-sectional study about the effects of work-related adverse events on the mental health of Dutch (resident) obstetrician-gynaecologists (ObGyns)

**DOI:** 10.1186/s12888-024-05678-3

**Published:** 2024-04-16

**Authors:** Melanie A.M. Baas, Claire A.I. Stramrood, Jolijn E. Molenaar, Petra M. van Baar, Joost W. Vanhommerig, Maria G. van Pampus

**Affiliations:** 1 Department of Obstetrics and Gynaecology, OLVG, 1090 HM Amsterdam, PO box 95500, The Netherlands; 2 Department of Obstetrics and Gynaecology, University Medical Center Groningen, 9700 RB Groningen, PO box 30.001, The Netherlands; 3 Beval Beter, 1000 AH Amsterdam, PO box 345, The Netherlands; 4 Amsterdam Reproduction and Development Research Institute, Amsterdam UMC, 1105 AZ Amsterdam, the Netherlands; 5 Department of Research and Epidemiology, OLVG, 1090 HM Amsterdam, PO box 95500, The Netherlands

**Keywords:** ObGyns, Adverse event, Second victim, Trauma, PTSD

## Abstract

**Background:**

Obstetrician**-**Gynaecologists (ObGyns) frequently face work-related adverse events such as severe obstetric complications and maternal or neonatal deaths. In 2014, the WATER-1 study showed that ObGyns are at risk of developing work-related posttraumatic stress disorder (PTSD), while many hospitals lacked a professional support system. The aim of the present study is to evaluate the current prevalence of work-related traumatic events and mental health problems among Dutch ObGyns, as well as to examine the current and desired support.

**Methods:**

In 2022, an online questionnaire was sent to all members of the Dutch Society of Obstetrics and Gynaecology (NVOG), including resident and attending ObGyns. The survey included questions about experienced work-related events, current and desired coping strategies, and three validated screening questionnaires for anxiety, depression, and PTSD (HADS, TSQ, and PCL-5).

**Results:**

The response rate was 18.8% and 343 questionnaires were included in the analysis. Of the respondents, 93.9% had experienced at least one work-related adverse event, 20.1% had faced a complaint from the national disciplinary board, and 49.4% had considered leaving the profession at any moment in their career. The prevalence rates of clinically relevant anxiety, depression, and psychological distress were 14.3, 4.4, and 15.7%, respectively. The prevalence of work-related PTSD was 0.9% according to DSM-IV and 1.2% according to DSM-5. More than half of the respondents (61.3%) reported the presence of a structured support protocol or approach in their department or hospital, and almost all respondents (92.6%) rated it as sufficient.

**Conclusions:**

The percentages of anxiety, depression, psychological distress and PTSD are comparable to the similar study performed in 2014. Most Dutch ObGyns experience adverse events at work, which can be perceived as traumatic and, in certain cases, may lead to the development of PTSD. Structured support after adverse work-related events is now available in almost two-thirds of workplaces, and was mostly experienced as good. Despite substantial improvements in the availability and satisfaction of professional support after work-related adverse events, the prevalence rates of mental problems remain considerable, and it is imperative to sustain conversation about the mental well-being of ObGyns.

**Supplementary Information:**

The online version contains supplementary material available at 10.1186/s12888-024-05678-3.

## Background

There is a growing body of evidence acknowledging physicians as a group with a high risk of work-related adverse events and posttraumatic stress disorder (PTSD), which has gained momentum as a result of the COVID-19 epidemic [1–3]. Physicians are frequently exposed to patients with severe illnesses, life-threatening emergencies, or the actual death of a patient. Obstetricians and gynaecologists (hereafter: ObGyns) are particularly vulnerable to a range of potentially distressing acute scenarios, such as fetal distress during labor, intrapartum fetal death, shoulder dystocia, unplanned caesarean section, maternal postpartum haemorrhage (PPH), and maternal or neonatal death [4–7]. Consequently, ObGyns are at higher risk of developing mental health issues, such as anxiety, depression, and work-related PTSD, compared to the general population [8, 9].

Several previous studies have reported that mental health issues are more common among physicians than the general population [10]. It is difficult to assess exact prevalence due to several factors: wide variation in diagnostic tests, use of self-reported measurements, and heterogeneity between populations. Nevertheless, crucial patterns cannot be overlooked. Studies examining anxiety disorders among physicians are scarce, but a Dutch study among several medical specialties showed a point prevalence of 13.6% versus a 12-month prevalence of 6.0% in the general population [9]. With regard to depressive disorders, international research has indicated up to a threefold higher depression rate [11] and double the suicide rate among physicians [12] in contrast to the general population. Among Dutch physicians, a point-prevalence of depression of 6.4% was observed compared to a 12-month prevalence of 6.1% in the general population [9].

Regarding PTSD, approximately 80.7% of the general Dutch population will experience at least one traumatic event during their lifetime, which is mostly not work-related, leading to a 7.4% development of PTSD at some point [13]. Despite the underrepresentation of physicians in PTSD research, a study among Dutch hospital physicians showed substantially higher rates of PTSD, with 20.8% experiencing traumatic events at the workplace, and 1.5% currently suffering from work-related PTSD [9]. A British study reported that two-thirds of ObGyns had been exposed to traumatic work-related events, with 17.9% reporting clinically significant PTSD symptoms [7]. In a Swedish study of 706 obstetricians, 70.9% experienced one or several severe events during their careers, after which 6.9% experienced symptoms indicative of PTSD [14]. Additionally, another study noted a more than two-fold prevalence of PTSD in physicians compared to the general population, with the highest PTSD prevalence found among ObGyns (18.0%) within the seven medical specialties studied [15]. It is plausible that the cultural context of the work of ObGyns varies globally, leading to different work experiences. Variations may include factors such as the volume of deliveries in a hospital, the proportion of planned caesarean sections, the frequency of obstetrical complications, funding sources for maternity units (private or public), and the ratio of skilled personnel to patients. Nevertheless, the inherent unpredictability of obstetrics and the potential of complications remain a shared factor globally.

In 2014, our research group conducted the WATER-1 study [8] to determine the prevalence of work-related traumatic events and PTSD among Dutch ObGyns, and to evaluate the adequacy of the support provided. Of the 683 respondents, 12.6% had experienced at least one work-related traumatic event and 1.5% met the criteria for PTSD. Of all respondents, 12.0% reported having a support protocol or strategy in their department or hospital, 25.7% were unaware of whether there was a protocol, and 62.3% reported that there was no such support. The support services after adverse events were rated insufficient by 60.0% at that time. These findings highlighted the urgent need to implement organized support.

The mental health of healthcare providers after exposure to adverse events is crucial for their overall well-being [16]. Physicians’ mental health may also affect patient safety by potentially leading to an increase in medical errors or defensive decision making [17–20]. Furthermore, healthcare providers suffering from psychological problems may show decreased productivity with burn-out or even career transitions [21–23], and incur collateral costs [24]. In 2015, the importance of healthcare professionals’ mental health was included as a new competency in the Canadian Medical Education Directives for Specialists 2015 (CANMEDS), a widely adopted framework defining the required skills for physicians working in healthcare [25]. In the Netherlands, the findings of the WATER-1 study were presented at the Gynaecongres (Dutch national gynecologists’ congress) in 2014, followed by focus groups to determine the optimal implementation of the study results. Consequently, the Dutch Society of Obstetrics and Gynaecology (NVOG) established the Committee for Collegial Support (CCO) in 2015, which provides support to ObGyns who have experienced adverse or traumatic events, and also offers assistance and support in case of formal complaints. In 2016, the Dutch Federation of Medical Specialists recommended the provision of professional support in all hospitals following medical incidents [26]. An abundance of scientific research shows that the provision of interventions aimed at reducing PTSD symptoms among healthcare providers, including offering professional support, is positively correlated with physician wellbeing [27–29]. Several years later, following the significant attention given to this topic and many local and national initiatives, we will continue to engage in ongoing developments around work-related adverse events and evaluate their effects.

Eight years after our initial study (WATER-1), the present study (WATER-2) was designed with three primary objectives. Firstly, it aimed to determine the current prevalence of anxiety, depression and psychological distress among (resident) ObGyns. Secondly, it aimed to explore adverse work-related events, subsequent coping strategies and support, and the prevalence of work-related PTSD. Lastly, it aimed to examine the accessibility of professional peer support (protocols) in the workplace and their perceived adequacy. Our hypotheses were that adverse work-related events still hold the potential for a traumatic event and may lead to PTSD. Furthermore, we hypothesized that most ObGyns currently have access to professional peer support in their workplace which they perceive as sufficient. 

## Methods section

### Design and participants

This study had a cross-sectional design. The participants were members of the NVOG, consisting mainly of resident and attending gynecologists in the Netherlands, but also of non-practicing and retired ObGyns. All 1825 registered members of the database were invited. Data were collected from the beginning of February to halfway through March 2022. An email containing the link to the questionnaire in the online survey system SurveyMonkey® was sent to all NVOG members. All participants were prospectively informed about the research and provided consent by agreeing with the first statement of the online questionnaire (“I give permission for using my answers (anonymously) for scientific purposes”). Personal data were not traceable or recorded from this email or the link to the webpage.

### Measurements

The 79-item questionnaire was inspired by a questionnaire used in a previous study [8], and further improved by our experience of using this questionnaire among other medical specialties [9] [9], aiming for the most accurate representation of reality. The questionnaire underwent updates, incorporating an additions set of questions (n=12) concerning aspects such as the COVID-19 pandemic, experience with the CCO, and number of traumatic events during their careers. Some questions (n=4) deemed irrelevant were removed, while certain topics were further elaborated by redistributing them across more questions (n=3). The sequence of questions was modified, and within individual questions, there were multiple instances of rephrasing, altering response formats, or introducing additions answer options. The survey was piloted among members of the CCO, after which minor changes were made, mainly clarified phrasing. The final questionnaire consisted of items on demographics (*n* = 5), personal experiences with work-related events (*n* = 10), impact of the COVID-19 pandemic (*n* = 2), respondents’ responses to work-related adverse events (*n* = 1), and actual and desired support after work-related adverse events (*n* = 7).

Furthermore, three validated self-report instruments were used in this study. The Hospital Anxiety and Depression Scale (HADS) was used to screen for anxiety and depression. This self-report questionnaire consists of two subscales: seven questions about the symptoms of depression (HADS-D) and seven questions about the symptoms of anxiety (HADS-A). These variables can be evaluated both separately (with a cutoff score of 8 or higher) and combined (with a cutoff score of 12 or higher) as a general measure of psychological distress [30, 31]. Psychometric properties have been validated with Cronbach’s alpha ranging from 0.67 to 0.90 (HADS-D) and from 0.68 to 0.93 (HADS-A) in different populations [30, 32].

Two validated screening questionnaires were used to screen for PTSD symptoms. The Trauma Screening Questionnaire (TSQ) is a validated 10-item screening instrument for evaluating PTSD symptoms according to the DSM-IV [33]. Psychometric properties were validated with Cronbach’s alphas ranging from 0.71 to 0.91 [34]. The cutoff value for provisional diagnosis of PTSD was 6 or higher.

The Posttraumatic Stress Disorder Checklist for DSM-R (PCL-5) is a validated 20-item self-report screener that assesses the 20 PTSD symptoms according to the DSM-5. A cutoff score of 33 or higher indicates probable PTSD. The psychometric properties are validated with a Cronbach’s alpha of 0.94 [35, 36].

In 2017, the new version of the Diagnostic and Statistical Manual of Mental Disorders (DSM-5) [37] was implemented in the Dutch healthcare setting. For PTSD, one of the most important differences between DSM-IV and DSM-5 was the removal of the A2-criterium regarding experiencing fear, helplessness, or horror during a traumatic event. In our previous study (WATER-1) in 2014, a screening instrument for PTSD was used that was based on the DSM-IV criteria (TSQ). To allow for (1) screening based on the most recent guidelines (i.e. DSM-5), we used the PCL-5 (2) an ‘eyeball-comparison’ between 2014 and 2022, we also used the TSQ in the current study.

In our study, respondents were asked whether they had experienced one or more traumatic events at work (one or more) at least four weeks ago. Those with an affirmative response (i.e., meeting DSM-5 criterion A) completed the PTSD Checklist for DSM-5 (PCL-5). Respondents who also reported experiencing fear, helplessness, or horror during the event (i.e., meeting DSM-IV criterion A2) completed the Trauma Screening Questionnaire (TSQ) in addition to the PCL-5.

### Statistical analysis

Statistical analyses were performed using IBM SPSS Statistics for Windows version 27. The multiple-choice questions and the 4-point Likert scale questions were analyzed using descriptive statistics. Chi-square tests and Fisher’s exact tests were used to compare subgroups for the categorical variables. All variables are presented as numbers and percentages. Multivariable logistic regression was used to assess the effects of function (attending vs. resident), sex (male vs. female), years of experience, and age on respondents’ mental health issues. Statistical significance was set at *p*-value of less than 0.05. A direct statistical comparison with the WATER-1 study [8] was not feasible because of two reasons. First, there were extensive changes in many questions of the questionnaire, including adding or removing questions, rephrasing questions, and adding or changing the answer options. Second, the respondent populations of 2014 and 2022 were partly overlapping, resulting in data being dependent rather than independent samples. Due to the anonymous questionnaire, this percentage of respondents was unknown.

## Results

A total of 343/1825 (18.8%) members of the NVOG completed the questionnaire. Respondents who started but did not complete the questionnaire (*n* = 35) were excluded from the final analysis. One respondent was a physician assistant and not an ObGyn, and was therefore not included in the analysis. The characteristics of the current respondents (*n* = 343) were comparable to those of the reference NVOG population (*n* = 1825, see Table 1), except for an underrepresentation of retired ObGyns in our sample compared to the membership database (Tables 1 and 2). Of all non-practicing ObGyns (*n* = 5), one respondent left the profession due to a work-related adverse event.

**Table 1:** Demographic characteristics of respondents and NVOG population

Variable	Respondents (*n* = 343)			NVOG population (membership database) (*n* = 1.825)	
	**n**	**(%)**		**n**	**(%)**
ObGyns					
Resident	84	(24.5)		419	(23.0)
Attending	229	(66.8)		1119	(61.3)
Non-practicing	5	(1.5)		**-**	**-**
Retired	25	(7.3)		287	(15.7)
Sex					
Male	90	(26.2)		614	(33.6)
Female	253	(73.8)		1211	(66.4)
Age					
25–34	61	(17.8)		-	-
35–44	117	(34.1)		-	-
45–54	79	(23.0)		-	-
55–64	53	(15.5)		-	-
≥ 65	33	(9.6)		-	-
Years in practice					
0–5	38	(11.1)		-	-
6–10	63	(18.4)		-	-
11–15	65	(19.0)		-	-
16–20	50	(14.6)		-	-
> 20	127	(37.0)		**-**	**-**

All variables are in number (%)

*NVOG* Dutch Society of Obstetrics & Gynaecology

-: Unknown data

**Table 2:** Demographic variables per subgroup of the respondents

	Total (*n* = 343)		Resident (*n* = 84)		Attending (*n* = 229)		Non-practicing (*n* = 5)		Retired (*n* = 25)	
	n	(%)	n	(%)	n	(%)	n	(%)	n	(%)
Sex										
Male	90	(26.2)	12	(14.3)	55	(24.0)	2	(40.0)	21	(84.0)
Female	254	(73.8)	72	(85.7)	174	(77.0)	3	(60.0)	4	(16.7)
Age										
25–34	61	(17.7)	58	(69.0)	3	(1.3)	0	(0.0)	0	(0.0)
35–44	117	(34)	25	(29.8)	92	(40.7)	0	(0.0)	0	(0.0)
45–54	80	(23.3)	1	(1.2)	78	(34.1)	1	(20.0)	0	(0.0)
55–64	53	(15.4)	0	(0.0)	52	(22.7)	2	(40.0)	0	(0.0)
≥ 65	33	(9.6)	0	(0.0)	4	(1.8)	2	(40.0)	25	(100)
Years in practice										
0–5	38	(11.0)	37	(44.0)	1	(0.4)	0	(0.0)	0	(0.0)
6–10	63	(18.3)	39	(46.4)	24	(10.6)	0	(0.0)	0	(0.0)
11–15	65	(18.9)	8	(9.5)	56	(24.8)	1	(20.0)	0	(0.0)
16–20	50	(14.5)	0	(0.0)	48	(21.2)	1	(20.0)	1	(4.2)
> 20	128	(37.2)	0	(0.0)	100	(43.7)	3	(60.0)	24	(96.0)
Work-related adverse event	320	(93.0)	78	(92.9)	209	(92.5)	5	(100.0)	24	(100.0)
Complaint at disciplinary board	69	(20.1)	1	(1.2)	50	(22.1)	1	(20.0)	15	(62.5)

All variables are in number (%)

### Anxiety, depression, and psychological distress

Of all responding ObGyns, 49 (14.3%) scored above the cutoff value for anxiety (Table 3). Resident ObGyns were more likely to have anxiety scores above the cut-off than attending ObGyns (aOR 0.68; 95% CI 0.46 to 0.99, *p* = 0.042, also corrected for sex). Those with up to five years of work experience were more likely to report clinically relevant anxiety than those with over 20 years in practice (aOR 0.51, 95% CI 0.30 to 0.85, *p* = 0.011).

The prevalence of depression was 4.4%, and no significant differences were found across sex, age and function by multivariable logistic regression analyses.

Of the responding ObGyns, 54 (15.7%) scored above the cut-off for increased psychological distress. Females were at a significantly higher risk of scoring above the cut-off value for psychological stress than males (aOR 1.70, 95% CI 1.16 to 2.48, *p* = 0.006).

**Table 3:** The measurements of mental health of Dutch resident and attending ObGyns in 2022

	Total (*n* = 343)		Resident (*n* = 84)		Attending (*n* = 229)	
	n	(%)	n	(%)	n	(%)
Clinically relevant anxiety^a^	49	(14.3)	17	(20.2)	26	(11.4)
Clinically relevant depression^b^	15	(4.4)	3	(3.6)	11	(4.8)
Psychological distress^c^	54	(15.7)	16	(19)	34	(14.8)
Using DSM-IV:						
- PTSD criterion A	45	(13.1)	10	(11.9)	30	(13.1)
- Probable PTSD^d^	3	(0.9)	0	(0)	2	(0.9)
Using DSM-5:						
- PTSD criterion A	127	(37.0)	47	(56.0)	111	(48.5)
- Probable PTSD^e^	4	(1.2)	0	(0)	2	(0.9)

All variables are in number (%)

^A^ Measured with HADS-A cutoff ≥ 8

^B^ Measured with HADS-D cutoff ≥ 8

^C^ Measured with HADS cutoff ≥ 12

^D^ Measured with TSQ cutoff ≥ 6

^E^ Measured with PCL-5 cutoff ≥ 33

Not included in this table: non-practicing and retired ObGyns

### The influence of the COVID-19 pandemic

Among all respondents, 53 (15.4%) ObGyns reported that the COVID-19 pandemic situation had influenced their ability to cope with work-related traumatic events. Furthermore, 23 (6.7%) respondents reported increased difficulty in accessing help or support during the COVID-19 pandemic.

### Leaving the profession

Half of the responding ObGyns (*n* = 170; 49.4%) had considered leaving their profession at some point during their careers, of which 30 (17.6%) respondents mention that they regularly contemplate quitting. The most frequently mentioned reasons for considering leaving the profession were a work-life imbalance, high workload, high responsibility, demanding work culture, and interpersonal conflicts with colleagues. Notably, 15 (8.8%) of the ObGyns that had considered leaving their profession reported considering quitting due to a work-related adverse event.

### Work-related adverse events

**Fig. 1:**
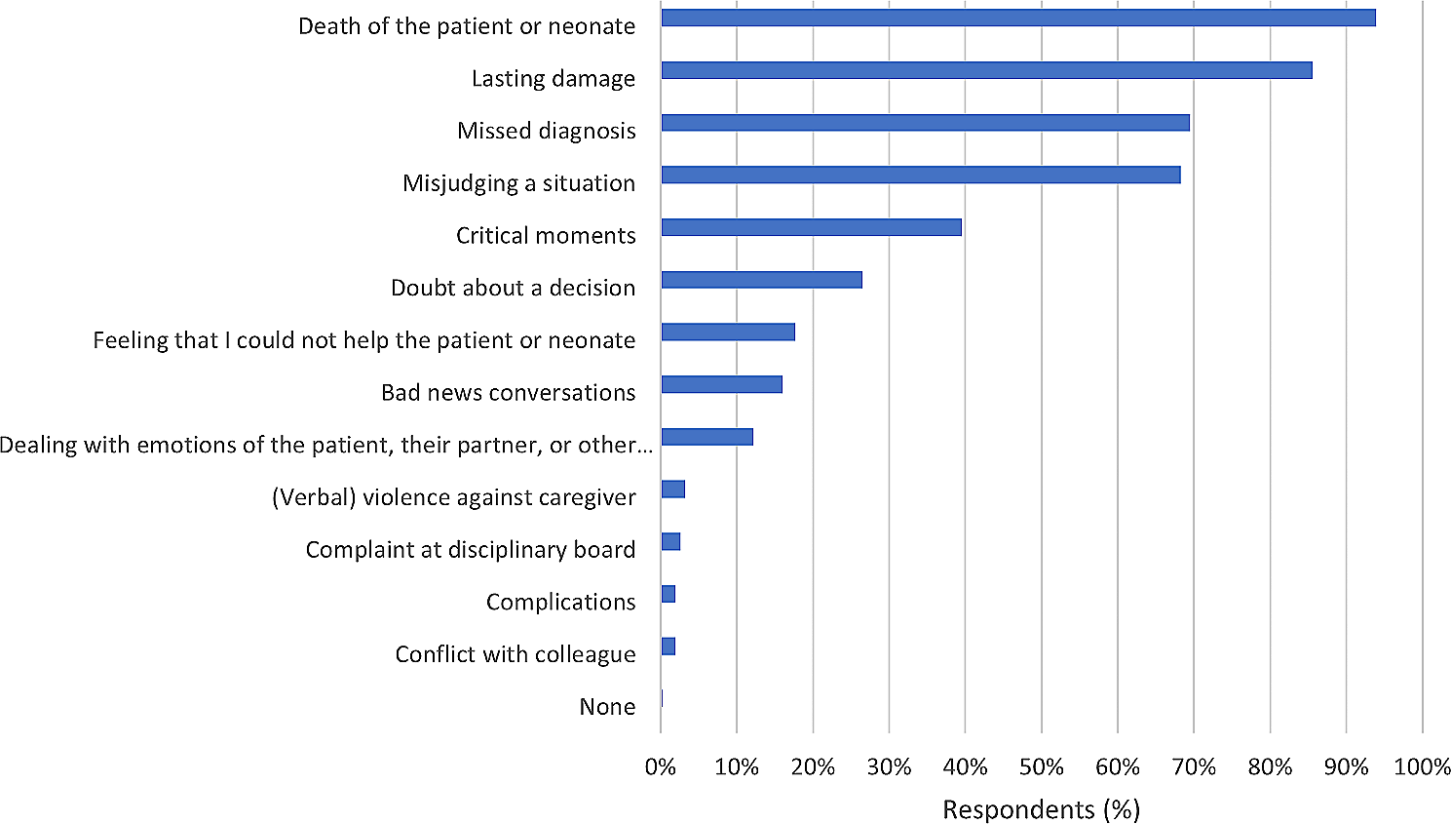
Events with high emotional impact (multiple answers possible)

Almost all respondents (*n* = 320; 93%) reported experiencing a work-related adverse event during their careers, which did not differ between resident or attending ObGyns (Table 2). The most commonly reported adverse events were death of the patient or neonate (93.9%), the knowledge that the patient or neonate will be left with lasting damage (85.5%), missing a diagnosis (69.5%) and misjudging a situation (68.3%) (Fig. 1). Attending ObGyns had a higher percentage of formal complaints (22.1%) compared to resident ObGyns (1.2%), with an average of 20.1% (Table 2). Of the ObGyns who faced a complaint at the disciplinary board (*n* = 69), 45 (65.2%) agreed that the disciplinary complaint had (unspecified) effects on their performance in the workplace.

### Posttraumatic stress disorder

Table 3 presents the outcomes of the TSQ and PCL-5 questionnaires. A higher percentage of ObGyns met the A-criterium for PTSD according to the DSM-5 criteria compared to DSM-IV (37.0% versus 13.1%), with 1.2% versus 0.9% meeting the criteria for PTSD.

### Coping

**Fig. 2:**
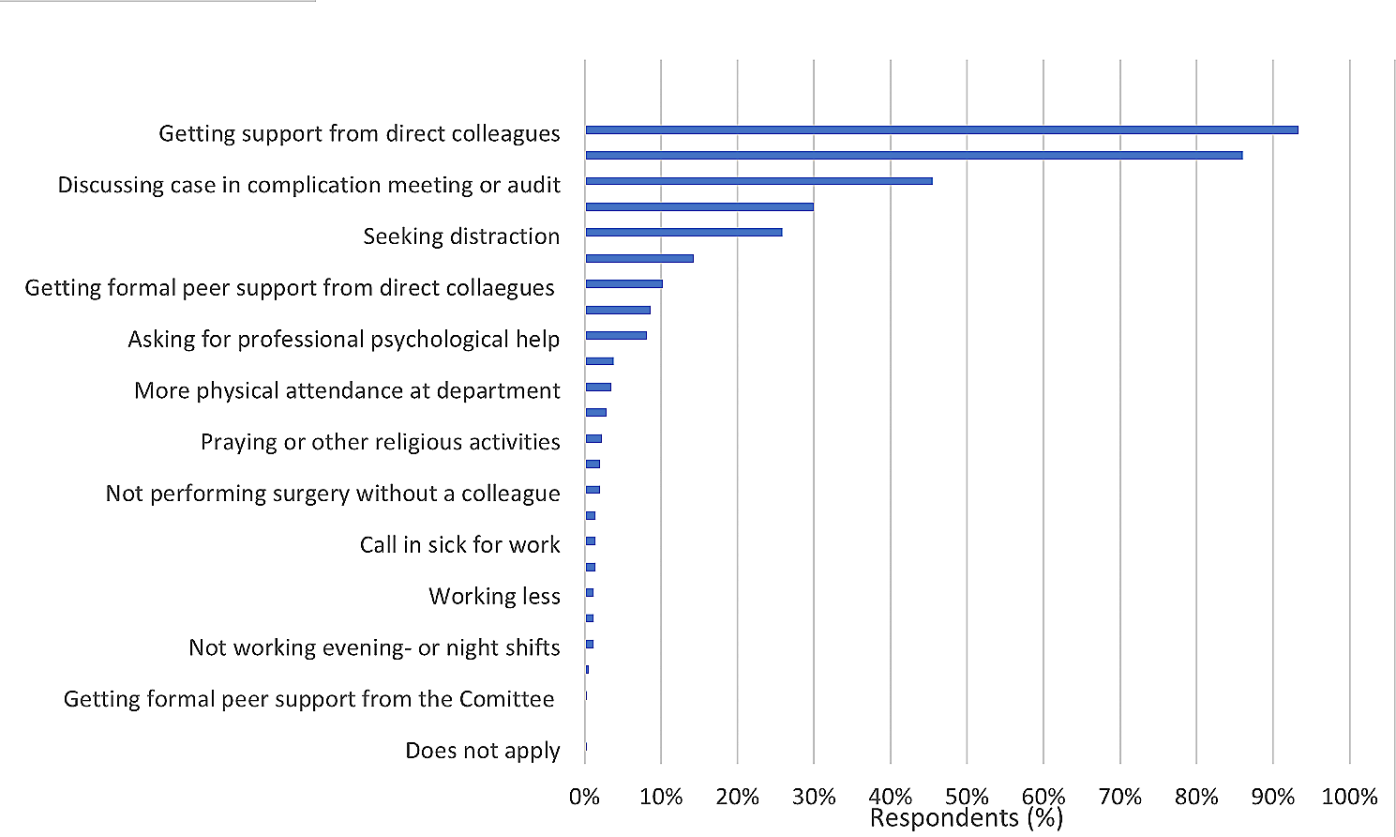
Coping strategies after a work-related adverse event (multiple answers possible)

The results regarding the coping strategies ObGyns use after a work-related adverse event are shown in Fig. 2. The most commonly used strategies were seeking informal support from direct colleagues (93.3%), talking with their partner, friends, or family (86.0%), discussing the case in a complication meeting or audit (45.5%), participating in sports or other hobbies (30.0%), and finding distraction in another way (25.9%).

### Formal support

More than half of the respondents (61.3%) reported the presence of a structured support protocol or approach at their workplace, 26.% did not know, and 10.5% reported there was no such protocol/approach. When a structured protocol/approach was present, this mostly consisted of professional peer support (43.6%), support from direct colleagues (37.8%), support from other colleagues in the department (29.9%), debriefing with involved caregivers (8.7%), team debriefing (6.4%), and guidance from a specialized care team (4.9%). Most respondents (92.6%) indicated that the currently offered support at the department was sufficient. The desired support was described as talking to a direct colleague (ObGyns) (29.8%), other colleagues in the department such as a nurse of midwife (21.1%), indirect colleagues from another hospital department (12.7%), and one-on-one meeting with a psychologist or coach (10.8%).

## Discussion

The results of this study show that ObGyns are not immune to mental health disorders. Additionally, the results support the hypothesis that adverse work-related events are (still) potentially traumatic and may lead to PTSD. Furthermore, the finding that the majority of ObGyns nowadays have access to professional peer support at their work place, and find it to be sufficient, aligns with our final hypothesis.

The **point** prevalence rates of anxiety and depression in the current study were 14.3% and 4.4%, respectively. These numbers are comparable to those of the WATER-1 study (15.8% and 6.5%), but compared to the **12-month** prevalences in the general Dutch population with a high educational degree (13.9% and 6.1%, respectively) [38], anxiety in particular appears to be higher. A higher prevalence among Dutch ObGyns compared to the general population is comparable with many previous literature that reported increased rates of anxiety and depression in medical students, resident doctors, and medical specialists compared to the general population [11, 39, 40]. Given the high prevalence of depression among physicians, and its association with a threefold increase in harmful medical errors [20, 41], this finding is of significant importance. In addition, in our study, females ObGyn were more likely to score above the cut-off value for psychological stress than their male colleagues. With the strong increase in female medical specialists the last decades, the field may need to evolve to ensure sustainable employability of their physicians. Offering peer support is a good starting point to anticipate the effects of traumatic exposure to work-related events, but PTSD is multifactorial. Incorporating the awareness of inevitable work-related adverse events should include a three-pronged approach. (1) Pre-trauma factors (such as prior psychopathology and prior or ongoing trauma) should be acknowledged and treated if indicated. There might be positive effects of training for healthcare professionals, but there is a need for more scientific evidence [42] (2) Trauma-related factors can only be changed in certain aspects of the situation, such as the supervision received and support provided directly after the event and peritraumatic emotional response or dissociation. (3) Post-trauma factors (cognitive processing of the trauma, social support, professional peer support, and social context (including methods or beliefs embedded in organizational structures [43]) is recommended in the education of ObGyns. While some of these factors are individual, others are organizational. With the results of our study, we aim for more awareness, both individual and organizational, of the intense emotional responses that might accompany adverse events. Our results support the current paradigm shift towards a safer workplace referred to as ‘just culture’, focusing on shared accountability and improving workplace systems instead of blaming the individual after adverse events [44].

In the period between the WATER-1 study and the current (WATER-2) study, the psychological consequences of adverse (or traumatic) work-related events for healthcare providers has emerged as an important topic in scientific research. The topic has gained interest worldwide, in specific the COVID-19 pandemic has heightened the need to understand the consequences of work-related traumatic events among healthcare providers. Although many healthcare workers might have experienced increased stress levels because of the COVID-19 pandemic [45], in the current study, a minority of 15.4% of all ObGyns stated that the situation around the COVID-19 pandemic influenced coping with work-related adverse events.

The current study revealed that the vast majority of respondents (93%) reported experiencing at least one work-related adverse event during their career, with 13.1 − 37.0% of these events classified as traumatic according to DSM-IV and DSM-5 criteria, respectively. The significant contrast in the application of DSM-IV versus DSM-5 is apparent, with nearly one-third of the traumatic events experienced by ObGyns meeting the criteria under DSM-5 but not being adequately recognized as traumatic using DSM-IV A criteria. Notably, the prevalence of traumatic events of 13.1% (WATER-2) when using the DSM-IV seems comparable to the 12.6% in the WATER-1 study [8].

In the current study, the prevalence rate of current PTSD was found to be 0.9– 1.2%, according to DSM-IV and DSM-5, respectively. The WATER-1 study reported a prevalence of PTSD of 1.3% according to DSM-IV, suggesting a comparable prevalence in recent times. These findings may indicate that respondents benefitted from enhanced professional support after adverse events and increased awareness in this area. However, given the limited numbers (with a maximum of 10 ObGyns with PTSD in the WATER-1 study) and the impossibility to make direct or longitudinal statistical comparisons, it is essential to interpret these results with caution. Although professional support after traumatic events is essential, it is also crucial to recognize the complexity surrounding PTSD. The development of PTSD depends on a range of elements such as genetic predispositions, personal coping mechanisms, pre-existing mental health conditions like depression or prior trauma and many others that collectively contribute to the susceptibility of developing PTSD. Lastly, the precise impact of the COVID-19 pandemic on our data remains uncertain. Although global evidence indicates a significant increase in PTSD prevalence among physicians as a result of the pandemic [42, 46], our study lacks the means to ascertain this influence.

In the present study, it was found that 61.3% of ObGyns reported having a structured protocol or approach for support at their workplace after being involved in an adverse work-related event, whereas only 12.0% of the ObGyns had such resources in the earlier WATER-1 study. The absence of any formal protocol or approach is reported by 10.5% of the respondents in the current study (WATER-2), which is substantially lower than the 62.3% reported in the earlier study (WATER-1). Additionally, 7.3% of respondents (WATER-2) found inadequate support (when available), which seems to be a considerable improvement from the 60.0% reported in the WATER-1 study. Collectively, these findings suggest a substantial enhancement in the accessibility and experienced quality of professional support following adverse events in the workplace.

The personal coping strategies employed by ObGyns appeared consistent with those identified in the WATER-1 study, predominantly involving seeking support from colleagues, partner, family, or friends. Interestingly, the current study indicated a higher proportion of respondents considering leaving the profession (49.4% in WATER-2 compared 33.7% in WATER-1). The most frequently cited reasons for this contemplating quitting included work-life imbalance and an excessive workload, with work-related adverse events accounting for this in a minority of cases (8.8%).

A notable strength of this study is its reevaluation of the consequences arising from adverse work-related events among Dutch ObGyns, employing a study design comparable to the WATER-1 study [8]. However, an important limitation is that a repeated measures study design with direct statistical comparisons was not feasible. This limitation stems from the partial overlap of respondents between 2014 and 2022, as the composition of the NVOG database changes over time. New members are added, resident ObGyns transition to attending ObGyns, attending ObGyns become non-practicing or retire, and some members unsubscribe). The database initially contained 1596 members in 2014, which increased to 1.825 in 2022. Additionally, the study encountered a lower response rate than in 2014 (18.2% versus 42.8%, respectively), though such response rates are common in survey studies. Another limitation of the study is the potential for selection bias which cannot be ruled out, as well as the unavailability of non-responder analysis due to the anonymous participation. Consequently, it was not possible to determine whether the prevalence rates of PTSD observed in our study were an underrepresentation or overrepresentation of the actual rates. Finally, it should be noted that only self-report questionnaires are used to estimate prevalences of anxiety, depression, and PTSD, whereas a clinical interview is required for a formal diagnosis.

In future research, a longitudinal design would be highly valuable, tracking resident ObGyns throughout their carriers to assess their mental well-being and their encounters with adverse work-related events. This approach enables comprehensive evaluation of psychological condition, including burnout symptoms and workload experiences. In addition, given that the PTSD literature among healthcare professionals is predominantly influenced by high-income Western countries, researchers in low- and middle-income countries or those with different cultural contexts are particularly encouraged to undertake studies focusing on the mental health of their ObGyns within their respective regions. One of the issues that emerged from the findings of the current study is what optimal support after work-related adverse events is. Despite a notable increase in satisfaction and availability of professional support in the workplace, a considerable number of ObGyns still reported a lack of sufficient support. Furthermore, even when professional support is accessible, it is crucial to acknowledge that healthcare providers may still encounter stigmas and barriers that hinder their access to adequate support [47].

Despite the current data suggesting substantial improvements in the availability and satisfaction of the professional support after work-related adverse events, the prevalence rates of anxiety, depression and PTSD remain considerable. Furthermore, the number of ObGyns contemplating leaving their profession continue to rise. Therefore, it is imperative to sustain a conversation about the mental well-being of ObGyns and maintain awareness of this critical issue in order to support their emotional health, improve retention within the profession, and ensure the quality of care for patients.

### Electronic supplementary material

Below is the link to the electronic supplementary material.

Supplementary Material 1

## Data Availability

The (online) questionnaire is attached (Additional file 1). Supporting data is not available in order to avoid that answers may be traced back to individual respondents.
