# Supplementary material for: Continuing the conversation: a cross-sectional study about the effects of work-related adverse events on the mental health of Dutch (resident) obstetrician-gynaecologists (ObGyns)
Source: BMC Psychiatry. 2024 Apr 16;24:286. doi: 10.1186/s12888-024-05678-3 (PMC11022402; doi:10.1186/s12888-024-05678-3)
Supplement: Supplementary file 1 — Supplementary Material 1 [file 12888_2024_5678_MOESM1_ESM.pdf]

Enquête WATER Gynecology II study

Inleiding

Welkom bij de vragenlijst over de impact van ingrijpende gebeurtenissen op de werkvloer.

Deze vragenlijst is onderdeel van de Work-related Adverse Traumatic Events Research (WATER) studies van de CAPTURE Group. Wij onderzoeken onder andere hoe de opvang is geregeld na het meemaken van ingrijpende gebeurtenissen op de werkvloer binnen verschillende medische specialismen.

Invullen van de vragenlijst duur ongeveer 15 minuten.

In 2014 inventariseerden wij voor het eerst de impact van ingrijpende gebeurtenissen op de werkvloer. In de afgelopen 8 jaar is er veel gebeurd, met name in de begeleiding na het meemaken van dergelijke gebeurtenissen. We willen graag opnieuw onderzoeken wat Gynaecologen meemaken en waar zij behoefte aan hebben.

Als arts kan men ingrijpende patiëntenzorg-gerelateerde gebeurtenissen meemaken. Bijvoorbeeld (dreiging van) overlijden of ernstig letsel van een patiënt, of bedreiging door een patiënt of familie.

Bij ingrijpende gebeurtenissen in deze vragenlijst gaat het over patiënt-gerelateerde gebeurtenissen, bijvoorbeeld medische situaties met een slechte afloop, calamiteiten, of overlijden. Het gaat expliciet niet over gebeurtenissen tussen collega's onderling, zoals problemen in de werksfeer of intercollegiale conflicten.

Als u tijdens het invullen van de vragenlijst terug wilt gaan naar de vorige pagina, gebruik dan [vorige] en niet de pijltjestoets boven aan de webpagina.

Alle antwoordgegevens zullen anoniem worden gebruikt ten behoeve van wetenschappelijk onderzoek.

* 1. Geeft u toestemming voor het (anoniem) gebruik van uw antwoordgegevens ten behoeve van wetenschappelijk onderzoek?

- [ ] Ja, ik geef toestemming

Enquête WATER Gynecology II study

Algemene gegevens

* 2. Geslacht

- Man
- Vrouw

* 3. Leeftijd

- 25-34 jaar
- 35-44 jaar
- 45-54 jaar
- 55-64 jaar
- 65 jaar en ouder

* 4. Hoeveel jaar bent u klinisch werkzaam (geweest) binnen de Gynaecologie/Obstetrie (inclusief werk als A(N)IOS)?

- 0-5 jaar
- 6-10 jaar
- 11-15 jaar
- 16-20 jaar
- Meer dan 20 jaar

* 5. Huidige functie

- AIOS
- Praktiserend Gynaecoloog
- Niet praktiserend Gynaecoloog (bijvoorbeeld management functie in de zorg, of een functie in een andere sector)
- Gepensioneerd
- Anders/eigen invulling:

* 6. Heeft het feit dat u niet praktiserend Gynaecoloog bent te maken met een ingrijpende gebeurtenis op de werkvloer?

Ja, de reden is:

Nee, de reden is:

7. Heeft u behoefte het hier nog over te hebben?

Ja, de reden is:

Nee, de reden is:

* 8. Wat is/was uw aandachtsgebied (meerdere antwoorden mogelijk)?

- [ ] Perinatologie
- [ ] Gynaecologische oncologie
- [ ] Benigne Gynaecologie
- [ ] Voortplantingsgeneeskunde
- [ ] Urogynaecologie
- [ ] (Nog) geen aandachtsgebied
- [ ] Anders/eigen invulling:

* 9. Heeft u ooit, om wat voor reden dan ook, overwogen te stoppen met het vak Gynaecologie/Obstetrie?

- ( ) Zeer vaak
- ( ) Regelmatig
- ( ) Wel eens
- ( ) Nooit

* 10. Is het overwegen te stoppen met het vak Gynaecologie/Obstetrie gerelateerd aan (een) ingrijpende gebeurtenis(sen) op de werkvloer?

- ○ Ja
- ○ Nee

* 11. Wat is de belangrijkste reden is dat u overwoog te stoppen met het vak Gynaecologie/Obstetrie?

12. Wilt u hier nog iets over kwijt?

* 13. Welke actie heeft u ondernomen met betrekking tot de overweging om te stoppen met het vak Gynaecologie/Obstetrie? (meerdere antwoorden mogelijk)

- ☐ Geen
- ☐ Ontslag genomen
- ☐ Met leidinggevende besproken
- ☐ Met collega's besproken
- ☐ Met partner, vrienden en/of familie besproken
- ☐ Naar andere carrière mogelijkheden binnen de Gynaecologie/Obstetrie gekeken
- ☐ Naar andere carrière mogelijkheden buiten de Gynaecologie/Obstetrie gekeken
- ☐ Anders/eigen invulling:

* 14. Ingrijpende gebeurtenissen op de werkvloer binnen de Gynaecologie/Obstetrie vind ik (meerdere antwoorden mogelijk):

- [ ] Geen
- [ ] Overlijden van een patiënt of neonaat
- [ ] Weten dat een patiënt of neonaat blijvende schade zal overhouden
- [ ] Het kritieke moment waarop een patiënt of neonaat in levensgevaar is
- [ ] Slecht nieuws moeten brengen
- [ ] Het over het hoofd zien van een diagnose
- [ ] Het verkeerd inschatten van een situatie
- [ ] Twijfelen of ik wel de juiste beslissingen neem
- [ ] Het gevoel hebben dat ik een patiënt of neonaat niet kan helpen
- [ ] Omgaan met emoties van de patiënt, partner of andere naasten
- [ ] Anders/eigen invulling:

* 15. Na het meemaken van een ingrijpende gebeurtenis tijdens mijn werk in de Gynaecologie/Obstetrie heb ik het volgende gedaan (meerdere antwoorden mogelijk):

- [ ] Niet van toepassing
- [ ] Niets
- [ ] Zo snel mogelijk naar huis gegaan
- [ ] Geprobeerd er niet aan te denken
- [ ] Afleiding gezocht, geprobeerd bezig te blijven
- [ ] Erover gepraat met partner, vrienden en/of familie
- [ ] Informeel erover gepraat met collega's
- [ ] Gesport of mij bezig gehouden met een hobby
- [ ] Mij ziekgemeld
- [ ] Minder gaan werken
- [ ] Geen (avond-/nacht-) diensten meer
- [ ] Meer fysiek aanwezig geweest op de verloskamers
- [ ] Geen vaginale stuitbevallingen meer
- [ ] Niet meer alleen geopereerd / sommige ingrepen niet meer alleen
- [ ] Meer dan normaal alcohol, drugs of sigaretten gebruikt
- [ ] Medicatie gebruikt die ik normaal zelden of niet gebruik
- [ ] Gebeden of mij tot andere religieuze activiteiten gewend
- [ ] Professionele psychische hulp gezocht
- [ ] Formele peer-support gevraagd aan directe collega's (eigen afdeling)
- [ ] Formele peer-support gevraagd aan indirecte collega's (andere afdeling)
- [ ] Formele peer-support gevraagd aan de NVOG Commissie Collegiale Ondersteuning
- [ ] Actie ondernomen gerelateerd aan patiëntveiligheid (complicatie-bespreking, perinatale audit, SIRE procedure, melding IGZ)
- [ ] Gestopt met werken in de Gynaecologie/Obstetrie
- [ ] Anders/eigen invulling:

* 16. Is er wel eens een klacht tegen u ingediend bij het tuchtcollege?

- ◯ Ja
- ◯ Nee

17. De tuchtzaak heeft effect gehad op mijn functioneren op de werkvloer.

- Helemaal mee eens
- Eens
- Oneens
- Helemaal mee oneens

18. Wilt u hier nog iets over kwijt?

COVID-19 pandemie

De volgende stellingen gaan over de situatie rondom de COVID-19 pandemie.

* 19. De situatie rondom de COVID-19 pandemie heeft voor mij invloed gehad op het verwerken van een ingrijpende gebeurtenis op de werkvloer.

- Helemaal mee eens
- Eens
- Oneens
- Helemaal mee oneens
- Niet van toepassing

* 20. De situatie rondom de COVID-19 pandemie heeft het lastiger voor mij gemaakt om hulp te zoeken.

- Helemaal mee eens
- Eens
- Oneens
- Helemaal mee oneens
- Niet van toepassing

Naar aanleiding van eerder uitgevoerd onderzoek bleek dat er behoefte was aan het krijgen van de juiste ondersteuning na het meemaken van een ingrijpende gebeurtenis op de werkvloer.

De volgende vragen gaan over uw ervaring met nazorg.

* 21. Op de afdeling/in de vakgroep waar ik werk is een vaste aanpak (denk aan een protocol of werkwijze, c.q. peer-support) voor nazorg na ingrijpende gebeurtenissen.

- Weet ik niet
- Niet van toepassing
- Nee
- Ja, dit bestaat uit:

* 22. Ik vind de huidige vorm van nazorg <u>op de afdeling/in de vakgroep</u> waar ik werk goed.

- Helemaal mee eens
- Eens
- Oneens
- Helemaal mee oneens

* 23. In het ziekenhuis/de kliniek waar ik werk is een vaste aanpak (denk aan een protocol of werkwijze, c.q. peer-support) voor nazorg na ingrijpende gebeurtenissen.

- ◯ Weet ik niet
- ◯ Niet van toepassing
- ◯ Nee
- ◯ Ja, dit bestaat uit:

* 24. Ik vind de huidige vorm van opvang na een ingrijpende gebeurtenis in het ziekenhuis/de kliniek waar ik werk goed.

- Helemaal mee eens
- Eens
- Oneens
- Helemaal mee oneens

* 25. <u>Op de afdeling/in de vakgroep</u> waar ik werk is ruimte voor emoties na het meemaken van een ingrijpende gebeurtenis.

- ◯ Helemaal mee eens
- ◯ Eens
- ◯ Oneens
- ◯ Helemaal mee oneens
- ◯ Niet van toepassing

* 26. Nadat ik een ingrijpende gebeurtenis op de werkvloer had meegemaakt bestond de opvang (c.q. peer-support) uit (meerdere antwoorden mogelijk):

- ☐ Opvang door collega’s op afdeling (bijv. verloskundige, verpleegkundige etc.)
- ☐ Opvang door directe collega’s (Gynaecologen / AIOS)
- ☐ Opvang door collega’s van een andere afdeling
- ☐ Opvang door aanbod vanuit het ziekenhuis
- ☐ Aanbod psycholoog of psychische hulp intern
- ☐ Aanbod psycholoog of psychische hulp extern
- ☐ 1-op-1 gesprekken met een psycholoog of coach
- ☐ Een buddy
- ☐ Niets
- ☐ Anders/eigen invulling:

* 27. Na het meemaken van een ingrijpende gebeurtenis zou ik graag opvang willen krijgen in de vorm van (meerdere antwoorden mogelijk):

- [ ] Opvang door collega's op afdeling (bijv. verloskundige, verpleegkundige etc.)
- [ ] Opvang door directe collega's (Gynaecologen / AIOS)
- [ ] Opvang door collega's van een andere afdeling
- [ ] Opvang door aanbod vanuit het ziekenhuis
- [ ] Aanbod psycholoog of psychische hulp intern
- [ ] Aanbod psycholoog of psychische hulp extern
- [ ] 1-op-1 gesprekken met een psycholoog of coach
- [ ] Een buddy
- [ ] Niets
- [ ] Anders/eigen invulling:

Enquête WATER Gynecology II study

Naar aanleiding van het eerder uitgevoerde onderzoek is de Commissie Collegiale Ondersteuning opgericht vanuit de Nederlandse Vereniging voor Obstetrie en Gynaecologie (NVOG).

Gynaecologen en A(N)IOS kunnen hier terecht voor onder andere peer support, er kan een luisterend oor worden geboden na het meemaken van ingrijpende gebeurtenissen, en er kan ondersteuning en informatie worden gegeven bij een tucht- of rechtszaak.

* 28. Kent u de Commissie Collegiale Ondersteuning?

- ○ Ja
- ○ Nee

* 29. Weet u hoe de Commissie Collegiale Ondersteuning te raadplegen of te vinden?

- Ja
- Nee

30. Zou u het overwegen bij een ingrijpende gebeurtenis contact op te nemen met de Commissie Collegiale Ondersteuning?

Ja, omdat:

Nee, omdat:

De volgende vragen gaan over hoe u zich voelt. Lees iedere vraag goed door en geef uw antwoord dat het beste weergeeft hoe u zich gedurende de afgelopen week gevoeld heeft.

Denk niet te lang na over een antwoord. Het gaat bij deze uitspraken om uw eigen indruk.

* 31. Ik voel me gespannen

- Meestal
- Vaak
- Af en toe, soms
- Helemaal niet

* 32. Ik geniet nog steeds van de dingen waar ik vroeger van genoot

- Zeker zo veel
- Wel wat minder
- Duidelijk minder
- Eigenlijk nauwelijks nog

* 33. Ik heb een soort angstgevoel alsof er iets vreselijks zal gebeuren

- Jazeker, vrij erg
- Ja, maar niet zo erg
- Een beetje, maar het hindert me niet
- Helemaal niet

* 34. Ik kan best lachen en de dingen van de vrolijke kant zien

- Net zoveel als vroeger
- Nu wel wat minder
- Duidelijk minder
- Helemaal niet

* 35. Ik maak me ongerust

- Heel erg vaak
- Vaak
- Af en toe maar niet te vaak
- Heel soms

* 36. Ik voel me opgewekt

- Helemaal niet
- Heel af en toe
- Soms
- Meestal

* 37. Ik kan rustig zitten en me ontspannen

- Jazeker
- Meestal
- Niet vaak
- Helemaal niet

* 38. Ik voel me alsof alles moeizamer gaat

- Bijna altijd
- Heel vaak
- Soms
- Helemaal niet

* 39. Ik krijg een soort angstig, gespannen gevoel in mijn buik

- Helemaal niet
- Soms
- Vrij vaak
- Heel vaak

* 40. Het interesseert me niet meer hoe ik eruit zie

- Inderdaad, helemaal niet meer
- Niet meer zoveel als eigenlijk zou moeten
- Het interesseert me wel, maar iets minder dan vroeger
- Het interesseert me net zoveel als vroeger

* 41. Ik ben onrustig en voel dat ik iets te doen moet hebben

- Inderdaad, heel duidelijk
- Duidelijk
- Enigszins
- Helemaal niet

* 42. Ik verheug me van tevoren op dingen die komen gaan

- Net zoveel als vroeger
- Een beetje minder dan vroeger
- Veel minder dan vroeger
- Bijna nooit

* 43. Ik raak plotseling in paniek

- Inderdaad, zeer vaak
- Tamelijk vaak
- Soms
- Helemaal nooit

* 44. Ik kan van een goed boek genieten, of van een radio- of televisieprogramma

- Vaak
- Tamelijk vaak
- Af en toe
- Zelden

Als arts kan men ingrijpende patiëntenzorg-gerelateerde gebeurtenissen meemaken. Bijvoorbeeld (dreiging van) overlijden of ernstig letsel van een patiënt, of bedreiging door een patiënt of familie.

* 45. Heeft u ooit tijdens uw werk soortgelijke situaties/gebeurtenissen meegemaakt?

- Ja
- Nee

* 46. Heeft/hebben deze gebeurtenis(sen) minimaal 4 weken geleden plaatsgevonden?

- Ja
- Nee

* 47. Reageerde u bij tenminste één van deze gebeurtenissen met intense angst, hulpeloosheid of afschuw?

- ◯ Ja
- ◯ Nee

Enquête WATER Gynecology II study

De volgende vragen gaan over uw persoonlijke reactie op de ingrijpende gebeurtenis die u heeft meegemaakt.

Geef aan (ja/nee) of u tenminste tweemaal in de afgelopen week het volgende heeft ondervonden.

* 48. Verontrustende gedachten of herinneringen aan de gebeurtenis die in uw hoofd opkomen tegen uw wil in

◯ Ja

◯ Nee

* 49. Verontrustende dromen over de gebeurtenis

◯ Ja

◯ Nee

* 50. U gedragen of u voelen alsof de gebeurtenis opnieuw plaatsvindt

◯ Ja

◯ Nee

* 51. Van streek raken door dingen die u aan de gebeurtenis herinneren

◯ Ja

◯ Nee

* 52. Lichamelijke reacties (zoals snelle hartslag, steen in de maag, zweten, duizeligheid) wanneer u herinnerd wordt aan de gebeurtenis

◯ Ja

◯ Nee

* 53. Moeilijk in slaap kunnen komen of te vroeg wakker worden

◯ Ja

◯ Nee

* 54. Geïrriteerdheid of woedeaanvallen

◯ Ja

◯ Nee

* 55. Moeite hebben met concentreren

- Ja
- Nee

* 56. Verhoogd bewustzijn van mogelijke gevaren voor uzelf en anderen

- Ja
- Nee

* 57. Gespannen zijn of schrikken van iets onverwachts

- Ja
- Nee

* 58. Het is mogelijk dat u bij bovenstaande vragen 'nee' invult wat betreft afgelopen week, maar dat u veel van bovenstaande symptomen in een eerdere periode in uw leven ervaren heeft na een gebeurtenis op werk.

- Nee, ik herken de symptomen niet
- Ja, ik herken minstens 6 symptomen uit een eerdere periode in mijn leven, WEL gerelateerd aan werk
- Ja, ik herken minstens 6 symptomen uit een eerdere periode in mijn leven, NIET gerelateerd aan werk

De volgende vragen gaan over problemen die mensen soms kunnen ondervinden na een ingrijpende gebeurtenis.

Leest u alstublieft elke omschrijving aandachtig door terwijl u denkt aan uw meest ingrijpende gebeurtenis op de werkvloer en geef aan in hoeverre u er in de afgelopen maand last van heeft gehad.

In hoeverre heeft u in de afgelopen maand last gehad van:

* 59. Regelmatig terugkerende, onaangename en ongewenste herinneringen aan de stressvolle gebeurtenis?

- Helemaal niet
- Een beetje
- Matig
- Nogal veel
- Extreem veel

* 60. Regelmatig terugkerende, onaangename dromen over de stressvolle gebeurtenis?

- Helemaal niet
- Een beetje
- Matig
- Nogal veel
- Extreem veel

* 61. Opeens het gevoel hebben of u gedragen alsof de stressvolle gebeurtenis daadwerkelijk opnieuw plaatsvindt (alsof u terug bent in de tijd dat de gebeurtenis zich afspeelde, en het opnieuw beleeft)?

- Helemaal niet
- Een beetje
- Matig
- Nogal veel
- Extreem veel

* 62. Erg van streek raken wanneer iets u aan de stressvolle gebeurtenis herinnert?

- Helemaal niet
- Een beetje
- Matig
- Nogal veel
- Extreem veel

* 63. Een sterke lichamelijke reactie hebben wanneer iets u aan de stressvolle gebeurtenis herinnert (bijvoorbeeld: hartkloppingen, moeite met ademhalen, zweten)?

- Helemaal niet
- Een beetje
- Matig
- Nogal veel
- Extreem veel

* 64. Het vermijden van herinneringen, gedachten of gevoelens die verband houden met de stressvolle gebeurtenis?

- Helemaal niet
- Een beetje
- Matig
- Nogal veel
- Extreem veel

* 65. Het vermijden van dingen die herinneringen zouden kunnen oproepen aan de stressvolle gebeurtenis (bijvoorbeeld: bepaalde mensen, plekken, gespreksonderwerpen, activiteiten, voorwerpen of situaties)?

- Helemaal niet
- Een beetje
- Matig
- Nogal veel
- Extreem veel

* 66. Moeite hebben met het herinneren van belangrijke delen van de stressvolle gebeurtenis?

- Helemaal niet
- Een beetje
- Matig
- Nogal veel
- Extreem veel

* 67. Sterke, negatieve overtuigingen hebben met betrekking tot uzelf, anderen of de wereld (bijvoorbeeld gedachten hebben zoals: ik ben slecht, er is iets vreselijk mis met mij, niemand is te vertrouwen, de wereld is door en door gevaarlijk?)

- Helemaal niet
- Een beetje
- Matig
- Nogal veel
- Extreem veel

* 68. De schuld geven aan uzelf of aan anderen voor de stressvolle gebeurtenis of de gevolgen daarvan?

- Helemaal niet
- Een beetje
- Matig
- Nogal
- Extreem veel

* 69. Sterke, negatieve gevoelens ervaren zoals angst, afschuw, boosheid, schuld of schaamte?

- Helemaal niet
- Een beetje
- Matig
- Nogal veel
- Extreem veel

* 70. Verminderde interesse hebben in activiteiten die u eerder graag deed?

- Helemaal niet
- Een beetje
- Matig
- Nogal veel
- Extreem veel

* 71. Afstand voelen tussen uzelf en andere mensen, of u vervreemd voelen van andere mensen?

- Helemaal niet
- Een beetje
- Matig
- Nogal veel
- Extreem veel

* 72. Moeite hebben om positieve gevoelens te ervaren (bijvoorbeeld: niet in staat zijn om u gelukkig te voelen of om gevoelens van liefde te hebben voor de mensen die u nabij zijn)?

- Helemaal niet
- Een beetje
- Matig
- Nogal veel
- Extreem veel

* 73. Prikkelbaarheid, woedeaanvallen, of u agressief gedragen?

- Helemaal niet
- Een beetje
- Matig
- Nogal veel
- Extreem veel

* 74. Teveel risico's nemen of dingen doen die u schade zouden kunnen toebrengen?

- Helemaal niet
- Een beetje
- Matig
- Nogal veel
- Extreem veel

* 75. “Superalert”, waakzaam of op uw hoede zijn?

- Helemaal niet
- Een beetje
- Matig
- Nogal veel
- Extreem veel

* 76. U nerveus voelen of snel schrikken?

- Helemaal niet
- Een beetje
- Matig
- Nogal veel
- Extreem veel

* 77. Moeite hebben met concentreren?

- Helemaal niet
- Een beetje
- Matig
- Nogal veel
- Extreem veel

* 78. Moeite hebben met inslapen of doorslapen?

- Helemaal niet
- Een beetje
- Matig
- Nogal veel
- Extreem veel

Einde vragenlijst

Bedankt voor uw deelname aan de vragenlijst.

Mocht u behoefte hebben aan contact met de Commissie Collegiale Ondersteuning, dan kunt u de contactgegevens vinden op de inlogpagina van de NVOG (Inloggen - NVOG).

79. Ruimte voor opmerkingen aangaande dit onderwerp of de vragenlijst.
